# Are bottom-up approaches good for promoting social–ecological fit in urban landscapes?

**DOI:** 10.1007/s13280-019-01163-4

**Published:** 2019-03-16

**Authors:** Johan P. Enqvist, Maria Tengö, Örjan Bodin

**Affiliations:** 1 Stockholm Resilience Centre, Stockholm University, Kräftriket 2B, 10691 Stockholm, Sweden; 2 Department of Environmental and Geographical Science, African Climate and Development Institute, University of Cape Town, Private Bag X3, Cape Town, 7701 South Africa

**Keywords:** Bottom-up approaches, Environmental governance, Global South, Network analysis, Social–ecological fit, Urban lakes

## Abstract

Bottom-up approaches are often presented as a remedy to environmental governance problems caused by poorly aligned social institutions and fragmented ecosystems. However, there is a lack of empirical evidence demonstrating how such social–ecological fit might emerge and help achieve desirable outcomes. This paper combines quantitative social–ecological network analysis with interviews to investigate whether bottom-up approaches in lake governance improve the fit. We study groups of residents seeking to improve management of a network of lakes in Bengaluru, India. Results show that 23 ‘lake groups’ collaborate in a way that aligns with how lakes are hydrologically connected, thus strengthening the social–ecological fit. Three groups founded around 2010 have mobilized support from municipal officers and introduced an ecosystem-based approach to lake management that recognizes their ecological functions and dependence on, the broader hydrological network. These groups have also changed how other lake groups operate: groups founded after 2010 are more collaborative and more prone to contribute to social–ecological fit compared to the older lake groups. This paper demonstrates the utility of a theoretically informed method for examining the impact of bottom-up approaches, which, we argue, is important for a more informed perspective on their relevance and potential contribution to urban environmental governance.

**Electronic supplementary material:**

The online version of this article (10.1007/s13280-019-01163-4) contains supplementary material, which is available to authorized users.

## Introduction

The ‘problem of fit’ refers to the failure of social institutions to adequately match or align with ecosystems’ spatial, temporal, or functional features and dynamics (Lee 1993; Folke et al. 2007; Galaz et al. 2008). Urbanization, a key driver of global environmental change and an unprecedented transformation of human societies worldwide (Elmqvist et al. 2013; UN-Habitat 2013; McPhearson et al. 2016), tends to exacerbate problems of fit by assigning land use and administrative boundaries according to societal dynamics rather than local ecology (Grimm et al. 2008; Andersson et al. 2017). This obstructs the governance^1^ of the urban green spaces that are needed to create more livable cities and, importantly, for the provision of ecosystem services that underpin the well-being of urban dwellers (Millenium Ecosystem Assessment 2005; Elmqvist et al. 2013).

The literature on how to enhance fit is growing (Cumming et al. 2006; Olsson et al. 2007; Rijke et al. 2012; Epstein et al. 2015; Kininmonth et al. 2015). Several scholars have advocated for participatory and collaborative approaches, where local actors can create institutions that are adapted to site-specific conditions, thereby enhancing the fit from the bottom-up (Brown 2003; Olsson et al. 2007; Galaz et al. 2008). While collaborative approaches are often advantageous, it should also be noted that collaborating with too many partners can drain resources that could have been used more effectively for other purposes (Bodin et al. 2016). There is usually a need to prioritize, because who you collaborate with, and how, will impact the outcome (Bodin et al. 2014; Sayles and Baggio 2017a). Previous research has shown that collaborations that emerge from partners having shared interests are more productive than when they are mandated (Sayles and Baggio 2017b). However, it is also important to study what effects collaborations have more broadly, especially for the management of more fragmented ecosystems. Furthermore, bottom-up approaches should not be advocated uncritically, and there remains a need for empirical evidence on and methodological tools to assess the ways in which participatory approaches might enhance fit (Guerrero et al. 2015). Cities are conducive for studying participation, since the concentration of people incentivizes approaches that harness resident’s capacity, rather than treat their presence as an obstacle for environmental governance (Svendsen and Campbell 2008; Ernstson and Sörlin 2009; Krasny and Tidball 2015).

This paper aims to build knowledge about how bottom-up processes might contribute to good social–ecological fit. We study a network of community-based groups in Bengaluru (formerly Bangalore), working to restore and protect the Indian city’s water bodies and the waterways connecting them. These initiatives have influenced lake management locally (Nagendra 2016; Enqvist et al. 2016), however, their impact on social–ecological fit in the broader landscape of interconnected lakes remains unknown. Our first objective is therefore to use these ‘lake groups’ to test recent findings that fit can be driven by self-organizing actors following a bottom-up approach (Guerrero et al. 2015). Secondly, we seek to understand the social processes through which such fit emerges, specifically focusing on leadership and innovation in the lake group network. Some groups have signed memorandums of understanding (MOUs) with municipal authorities, giving them increased influence over and responsibility for lake management. These partnerships mean that this specific case represents a hybrid form of bottom-up governance, not only initiated from the bottom but also enabled by a permissive municipal government. The success of such public–civic collaborations has given these MOU groups “a sphere of influence well beyond their lakes, working with groups across the city to share their knowledge and experiences” (Nagendra 2016, p. 183). To operationalize and measure fit, we use an interdisciplinary framework that models lakes and associated lake groups as a network of ecological and social nodes connected by water flows and collaboration between groups (Bodin and Tengö 2012; Bodin et al. 2016; Sayles and Baggio 2017a). Previous studies of such networks have defined good fit by identifying patterns or ‘building blocks’ where social institutions and ecological features are well aligned (Bodin et al. 2014; Guerrero et al. 2015; Kininmonth et al. 2015; see Online Appendix S1 for details). We use this approach to quantitatively study whether lake groups’ work and interactions represent a good fit with the lake network, particularly the role of MOU groups in relation to older and newer initiatives. Further, qualitative information from interviews with each group is used to interpret these results and better understand how fit emerges and spreads in bottom-up initiatives. Three hypotheses guide this investigation:

### H1

The network displays good social–ecological fit. Bengaluru’s lake groups collaborate in a pattern that corresponds to good fit with the ecological network of interconnected lakes.

### H2

Fit stems directly from MOU groups. Lake groups with MOUs make a substantial contribution to the social–ecological fit of the network as a whole.

### H3

Fit stems from groups that are influenced by MOU groups. Lake groups without MOUs, but with links to a group with an MOU, make a substantial contribution to the social–ecological fit of the network as a whole.

This study employs a social–ecological network approach (Bodin and Tengö 2012) together with qualitative data to interrogate how bottom-up processes might influence environmental governance. Importantly, such initiatives are not always effective or preferable, so it is imperative to develop and test tools that can determine how they work in practice and how participatory management aligns with theories about ecosystem dynamics. Bengaluru is a relevant case study for two reasons: first, South Asia (and Sub-Saharan Africa) is projected to see most of the planet’s urban expansion, which is likely to disrupt, fragment, and complicate management of urban ecosystems that provide services to millions of residents (Seto et al. 2011; Fragkias et al. 2013; McDonald et al. 2013). Second, it is increasingly clear that cities in the global South often develop along different pathways than in the North; in particular, it is more common that residents rely on informal actors, infrastructure, and politics for provision of basic services such as water (Kudva 2009; Kooy 2014; Millington 2018). The citizen-based initiatives to protect and manage Bengaluru’s fragmented lake network, partially in collaboration with municipal actors, provide an excellent case for exploring how these two issues intersect.

## Materials and methods

### Eroded social–ecological fit in an urbanizing landscape

**Fig. 1:**
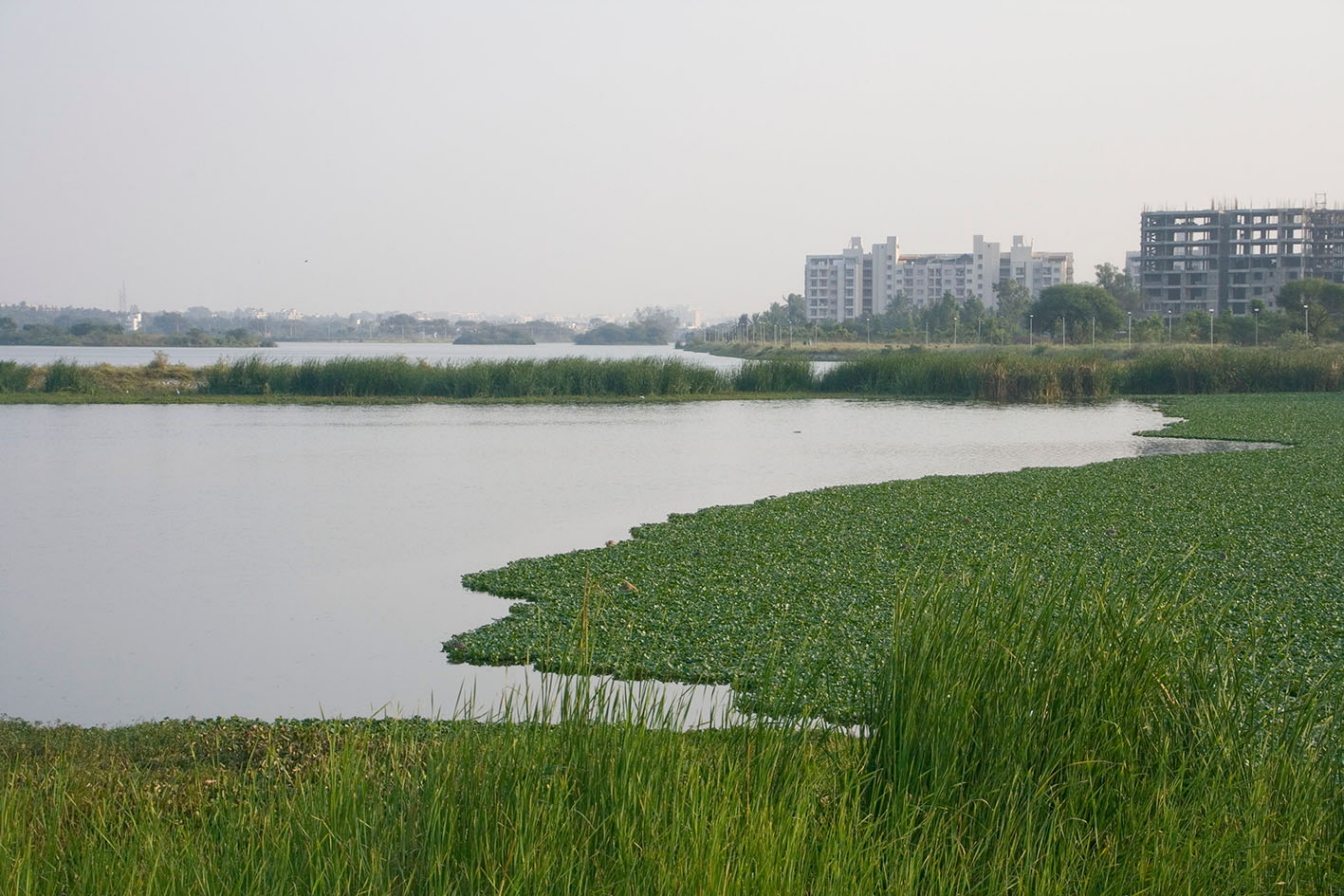
Jakkur Lake in the northern periphery of Bengaluru where large water bodies can still be found in relatively good condition. As in many other lakes in the city, increased effluent inflows from new residential developments with ineffective sewage treatment increase nutrient content and allow a layer of water hyacinth to spread (photo by the lead author)

India’s urban economies are expected to grow faster 2019–2035 than anywhere else on the planet, and Bengaluru is at the forefront of this development (Holt 2018). The UN World Urbanization Prospects (United Nations 2018) estimates the city’s population at 11.4 million, and growing rapidly due largely to the city’s booming IT and communications industry (Sudhira et al. 2007). Like many cities undergoing rapid development, Bengaluru’s water supply faces crucial challenges (McDonald et al. 2011, 2014; Lele et al. 2013). While poverty persists, economic growth has expanded the middle class and increased consumption, including demand on water supply (Fig. 1). Located in the semiarid plateau of south India, dry seasons in this area used to be endured by relying on water from reservoirs created by damming small, perennial streams. Farming villages had designated communities and individuals responsible for maintaining bunds, clearing silt and regulating flow from local lakes to store rainwater and provide for water needs throughout the year (Srinivas 2001; Nagendra 2016). For some lakes, these practices continued until the 1960s when all public land (including lakes) became state property (D’Souza and Nagendra 2011). Rapid urbanization during subsequent decades left both lakes and other urban ecosystems suffering from intensified use, expansion of built-up structures, and dumping of industrial and domestic wastes (Lele et al. 2013).

Today, the previous locally based management has been replaced by complex and overlapping jurisdictions amongst municipal and state authorities responsible for various water-related issues (D’Souza and Nagendra 2011; Nagendra and Ostrom 2014; Unnikrishnan and Nagendra 2014). The topography of the city divides lakes into three main watersheds (Central Ground Water Board 2008), but instead of organizing management according to hydrological connections, the municipality (*Bruhat Bengaluru Mahanagara Palike*, BBMP) assumed jurisdiction over lakes in central districts, while Bangalore Development Authority (BDA) manages most lakes in the more rapidly developing periphery (STUP Consultants Pvt. Ltd. 2011). This represents an archetypical case of social–ecological scale mismatch where governance authority is not aligned with the hydrological–geographical connectivity of the managed ecosystems (Cumming et al. 2006).

Many lake ecosystems have degraded or disappeared, impeding surface water regulation (flood mitigation) and groundwater availability (Nagendra and Ostrom 2014; Enqvist et al. 2016). Several community-based groups have formed to protect and restore a local lake, often seeking advice from ecologists and technical experts to adapt lake restorations to local conditions and emphasize the multiple functions they serve. These include recreation, biodiversity hotspots, livelihoods of less affluent communities, health and microclimate benefits, cultural, and religious events, and, importantly, recharging groundwater levels (Srinivas 2001; Nagendra et al. 2011; Unnikrishnan and Nagendra 2014; Enqvist et al. 2016). These groups have emerged in several different parts of the city (Enqvist et al. 2016) meaning that groups that coordinate their activities could create a social network of managers that aligns spatially with the network lakes in the city. They therefore present an important opportunity to empirically document the process of building fit from the bottom-up, which is a critically under-researched area (Guerrero et al. 2015 is an exception).

### Studying bottom-up initiatives

**Fig. 2:**
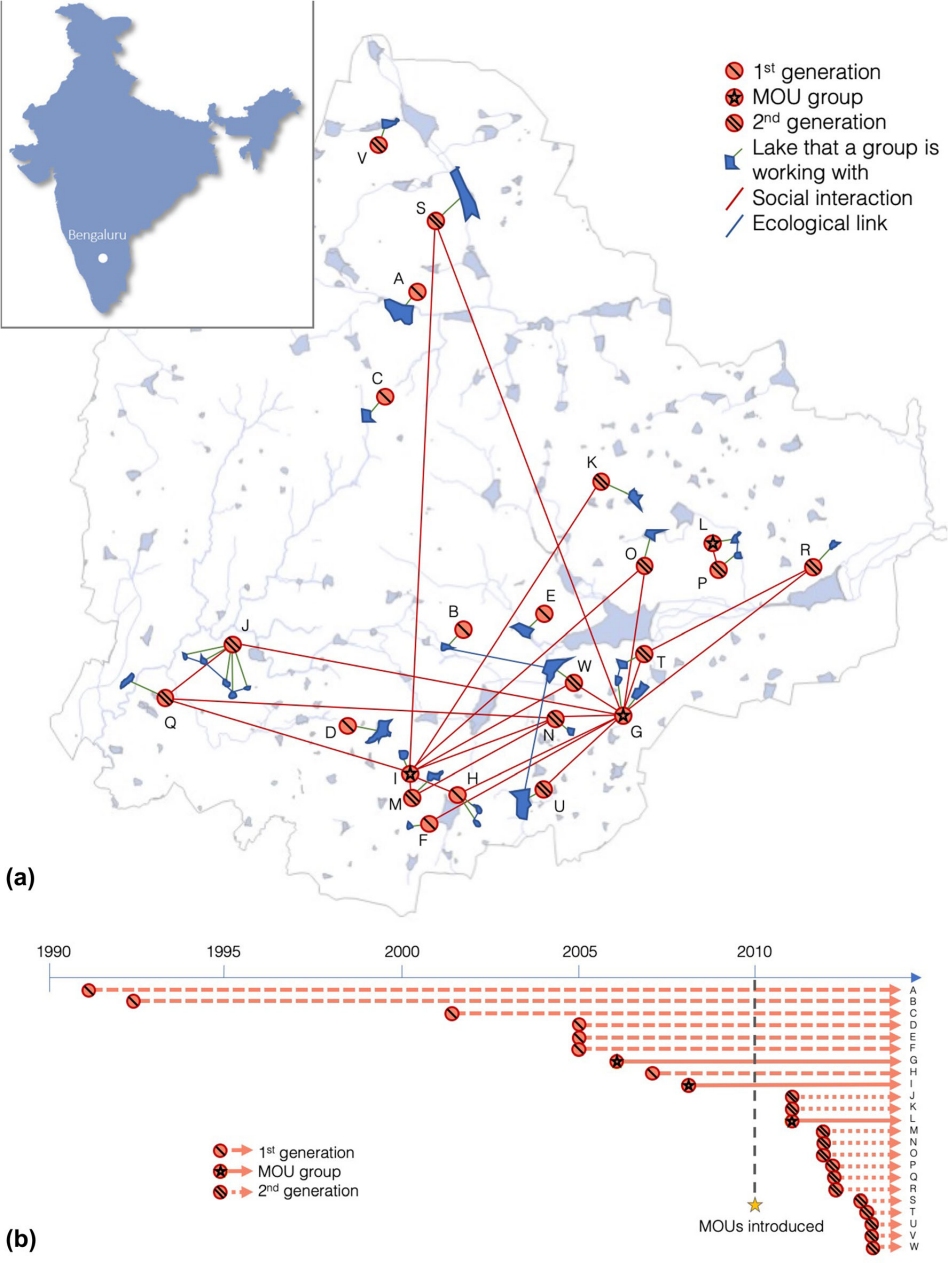
Lake groups in Bengaluru. Twenty-three lake groups (A–W) working with 29 lakes were studied. Location of lakes and the connections between them and lake groups are shown below (1.a). The timeline (1.b) shows approximately when the different groups first started actively working with a lake

Lake groups were identified through press reports, social media, lake activism platforms, and snowball sampling of key informants. We focused on “active” lake groups, those mobilizing local citizens and/or seeking to influence authorities to achieve change. Twenty-three lake groups met that criterion (indicated in Fig. 2 by letters A–W), covering a total of 29 lakes. While this only represents about 15% of the city’s water bodies, we deem it unlikely that a significant number of lake groups were omitted (for details on group selection and verification, see Enqvist et al. 2016). Since our objective was to investigate the implications for social–ecological fit from citizen involvement, specifically for the lakes they work with, we only included these 29 lakes in the network analysis.

In addition to three groups with MOUs, there are seven “1st-generation” groups founded before MOUs were introduced in 2010, and thirteen “2nd-generation” groups founded after 2010. The lead author conducted semistructured interviews and group interviews with up to five representatives from each group in October–December of 2013. Interviews covered how and why interviewees got involved with the groups; their collaborations with other actors and groups; their relation to the custodian authority in question; and the role of lake groups in the city’s continued development.

### Social–ecological network analysis

Our social–ecological network represents the Bengaluru landscape of human actors (lake groups) and natural elements (lakes) modeled as social and ecological nodes, that may or may not be connected through collaborations or water flow (Online Appendix S1). We identified ecological links using a hydrological map prepared for Bengaluru’s Lake Development Authority (STUP Consultants Pvt. Ltd. 2011). Social links between groups were defined as at least one group reporting recent interactions for the purpose of improving lake management, such as exchange of information or advice and/or visits to each other’s lakes. Any two groups that shared at least one active core member were also considered linked.

In the studied network, particular configurations of group collaborations and ecological connections are identified ‘building blocks’ (Bodin and Tengö 2012; Bodin et al. 2016) that can be theoretically associated with social–ecological fit or misfit (Table 1). We compared the frequencies of ten building blocks in the observed network with a null model based on 1000 randomized networks of equal size and structure, generated with the software package “Social–Ecological Network Analyzer (SENAnalyzer)” (Stockholm Resilience Centre 2019). We analyzed how often different types of groups were present in the various blocks, and what position in them they occurred in (see Online Appendix S1). Normally, Multi-Level Exponential Random Graph Model for network analysis would be more statistically robust, but since this statistical model does not explicate individual actors and all their positions in the various building blocks, it was less applicable for our study (Bodin et al. 2016).

**Table 1:**
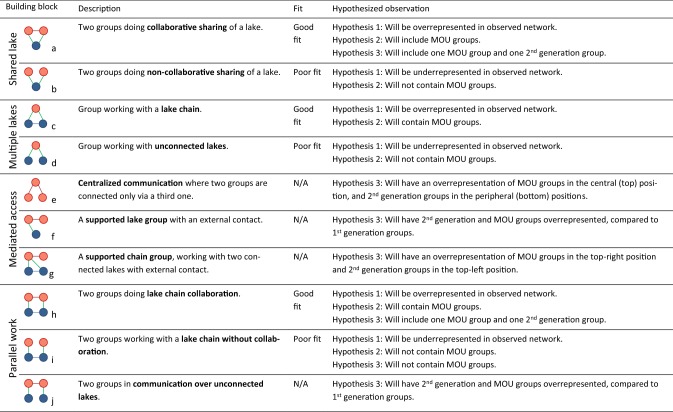
Building blocks studied in the analysis, and expected observations for each of the paper’s hypotheses: The network displays good fit (H1); Good fit is due to MOU groups’ positions (H2); Good fit is due to MOU groups’ influence on other lake groups (H3)

Table 1 summarizes how we use ten building blocks to test our three hypotheses, and if they indicate good or poor fit. For instance, a high prevalence of building block *c* indicates good fit—supporting Hypothesis 1—since it indicates that lake groups are more likely to work with lakes that are connected. If MOU groups are found in this position, this would also support Hypothesis 2. The building blocks are organized in four categories: *shared lake*, capturing cases where two groups work with the same lake; *multiple lakes*, indicating that one group works with at least two lakes; *mediated access*, where two groups are connected but only one of them also has a link to a lake, lake chain or a third group; and *parallel work*, where two groups work with one lake each.

Based on previous studies documenting the influential role of MOU groups on subsequent initiatives (Enqvist et al. 2016; Nagendra 2016), we expect to see MOU groups centrally located in the social network, linked primarily to 2nd-generation groups—especially in cases where this would contribute to better fit, e.g., by supporting lake chain groups (building block *g*) and participating in lake chain collaborations (building block *h*). This would support Hypothesis 3.

## Results

### Hypothesis 1: The network displays good social–ecological fit

**Fig. 3:**
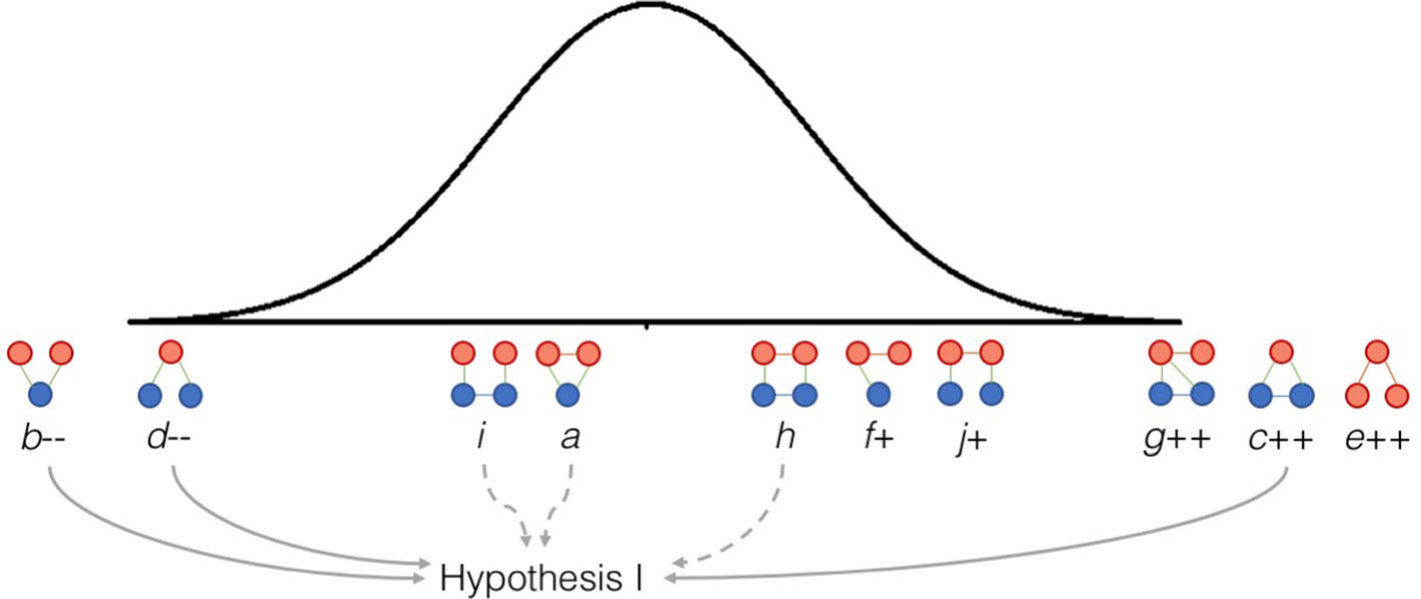
The presence of studied building blocks (*a*–*j*) compared to the normal distribution from 1000 randomized networks, represented by the bell curve (see Table A2.1 in Online Appendix S2 for details). Deviations from the null model are indicated with ++/−− for strong significance (*p* value < 0.01) and +/− for weak significance (*p* value < 0.05). No significant difference was found for building blocks *i*, *a*, and *h*. Arrows indicate what building blocks inform Hypothesis 1; solid lines show findings supporting the hypothesis, and dashed arrows show findings that neither support nor disprove it

‘Lake chain groups’ (building block *c*) occur significantly more often in the observed network than expected by chance (Fig. 3), which indicates a good fit (hypothesis 1, Table 1). Two building blocks that would indicate poor fit are almost absent: ‘non-collaborative sharing of lakes’ (building block *b*) and ‘groups working with unconnected lakes’ (building block *d*). Building blocks *a*, *h*, and *i* do not deviate from the null model, and hence, neither support nor reject the idea of good fit. The strongly significant deviation for three building blocks gives support for Hypothesis 1—there is a reasonably good fit.

It should be noted that since ‘lake chain collaborations’ are not overrepresented (building block *h*; Fig. 3), we can conclude that the observed fit is primarily a result of the way individual lake groups choose to work with lakes, rather than a tendency among groups to collaborate in ways that increase fit.

### Hypothesis 2: Fit stems directly from MOU groups

**Fig. 4:**
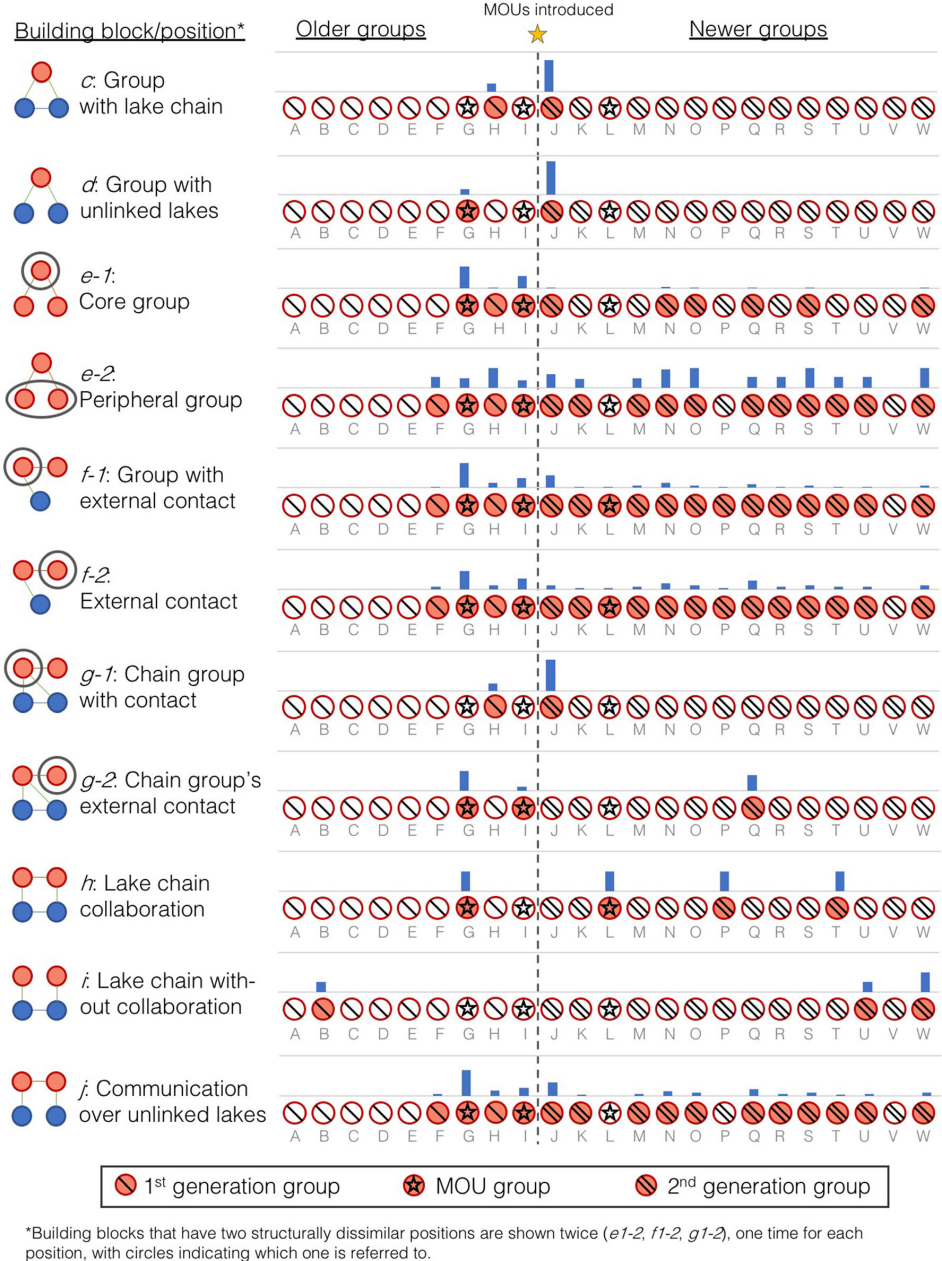
The presence of individual lake groups in studied building blocks. Each row focuses on one building block or building block position. The circles represent the 23 lake groups (A–W), indicating which ones occur in a specific building block (filled circles) or are absent (empty circles). Groups are ordered left-to-right according to how long they have been active, with the dashed line separating groups starting before and after MOUs were introduced. Bars above the circles indicate what groups occur most in each building block/position (normalized for each row, i.e., not comparable between rows). For example, for building block c, group J occurs four times as often as group H. Building blocks *a* and *b* are not included in this figure since they were not observed in the network

Results indicate that MOU groups (groups G, I, L) have an ambiguous relationship with fit. Contradicting the hypothesis (Table 1), the positional analysis (Fig. 4) shows that MOU groups do not increase fit by ‘working with lake chains’ (building block *c*)—in fact, group G increases misfit by ‘working with unconnected lakes’ (building block *d*). An interviewee from this group recounted how after restoring their first lake, they chose to work with a neighboring lake because it is situated in an active community. Group G subsequently decided that any future expansions should focus on lakes up- or downstream from their current areas of operation. This is reflected in group G’s support of the more recently formed group T, which works with a downstream lake—forming one of the network’s two ‘lake chain collaborations’ (building block *h*; Fig. 2a).

Both times ‘lake chain collaborations’ (building block *h*) appear they involve an MOU group. This means that although MOU groups do not themselves contribute to better fit by working with ‘lake chains’ on their own (building block *c*), we still find some support for Hypothesis 2 in that there is always an MOU group involved when multiple groups team up to protect interconnected lakes.

### Hypothesis 3: Fit stems from groups that are influenced by MOU groups

‘Groups working with lake chains’ (building block *c*) are the most important for creating good fit in the network. Our results show that all of these groups have an external support (building block* g*) from at least one MOU group; in fact, 60% of the times this building block occurs, position* g*-*2* is held by an MOU group (Online Appendix S2). Interview findings strengthen this connection: the remaining 40% all consist of links from a newer group reporting an interaction with the lake chain group, rather than the opposite (see group Q and group J; Fig. 2). All interactions reported by lake chain groups are with more senior MOU groups, meaning that the groups that do the most to improve social–ecological fit always do so with support from at least one older MOU group.

As hypothesized, MOU groups are also influential in the network more broadly: ‘centralized communication’ (building block *e*) is strongly overrepresented in the network (Fig. 2), and the central position (*e*-*1*, Fig. 3) is occupied by MOU groups G and I, 71 of 82 times (see Online Appendix S2). The peripheral position (*e*-*2*) is in most cases occupied by 2nd-generation groups, which means that most of the social interaction in the network occurs between MOU groups (G and I) and groups formed later than them.

Compared to other lake groups, Fig. 3 shows that 1st-generation groups are rarer in building blocks indicating communication, such as ‘supported lake groups’ (building block *f*) and ‘communication over unconnected lakes’ (building block *j*). A possible explanation could be that once groups are more established, they need less information and support; however, interview findings indicate that 1st-generation groups simply were not aware of many other lake groups, if any. Newer groups interact with others more often, and usually do so with at least one MOU group. This link is also visible in the two ‘lake chain collaborations’ (building block *h*), which both include an MOU group and a 2nd-generation group. A member of a newer group captures the nature of these interactions:

We knew who the authorities are. But that was only in principle, because that information is available anywhere. What [group L] helped me, us with was [knowing the] attitude [of the relevant officer], the kind of person, his approachability, […] Definitely, we are thankful […] that [group L’s] experience is of use to us, especially towards the beginning part of it when we launched. […] They made our journey a little easier.

*Group P*

In summary, we find that the results support Hypothesis 3: MOU groups influence others to contribute to good fit. They are always present in some capacity when a series of connected lakes are being worked on, either by supporting groups that work with lake chains on their own (building block *g*) or by being the “senior” partner in a ‘lake chain collaboration’ (building block *h*). Importantly, all three MOU groups do at least one of these two things, and not a single one of the other 20 groups do either of them.

### Processes of change in social–ecological fit

The results described above indicate that the MOUs introduced in 2010 changed how local actors work with lakes. New lake groups and collaborations emerge in a pattern that indicates that the way in which water flows between lakes influence what lakes a group starts working with, and what existing groups it reaches out to for help. In addition to this, we further highlight three processes that emerged from our qualitative interviews as drivers of this change: increased communication, spreading engagement, and improved relations with public authorities. We note that none of the processes necessarily leads to good fit in and of itself, but in our case, they help tell the story of how fit improved.

First, newer groups communicate to a higher extent than older ones. Less than 30% of 1st-generation groups interact with another group (compared to over 92% of the 2nd generation). The ones that do are the newest ones, and their contacts are the first MOU groups (G and I), started around the same time. Groups G and I act as centrally located “hubs” in the network, connecting many other groups that are not linked to each other (Fig. 4, position *e*-*1*), thereby contributing significantly to overall collaboration among lake groups.

Second, lake engagement is spreading. Nine of the groups started in the 20 years before MOUs were introduced in 2010, and 14 emerged in the 3 years after that (Fig. 2b). New groups often refer to MOU groups as “success stories” to learn from and be inspired by them:

When we go [to get people to support our group] we first show the success stories. That makes the people [realize]: “Okay, if it’s possible there, why not here?” That’s the reason many people have joined us.

*Group O*

The third process driving change is the improved relationship between lake groups and public agencies. For instance, four out of seven 1st-generation groups are or have been involved in legal processes against public authorities, but no other groups have adopted this approach. Instead, they typically advocate for the local community to become more involved and collaborate with the relevant authorities. Several interviewees also describe changing attitudes within the municipality:

I don’t think BBMP was so approachable before. It was very risky. We couldn’t talk to the [local political representative]. Now, even they are scared—of citizens. Because [citizens] are getting active, and I think a lot of gutsy people have stepped in. […] Before, filing [a request for public records] was considered risky—people would be targeted.

*Group J*

Local actors have certainly played crucial roles in creating the MOU approach, but it would not have happened without unconventional authority representatives agreeing to cede some power. The timeline in Fig. 2b clearly shows that it was not after groups G and H were formed (in 2006 and 2008, respectively) that lake groups started emerging more rapidly, but after the introduction of MOUs in 2010. Government representatives are therefore important enablers of grassroots mobilization by providing acknowledgment and support from above.

## Discussion

This paper shows that residents’ initiatives contribute positively to social–ecological fit between lakes and lake management in Bengaluru. By differentiating between older and newer groups, and between those operating on their own and those formally recognized in MOUs with municipal authorities, our analysis also describes how this fit emerged, and the key roles supporting it. Taken together, this provides an important demonstration of how to examine what potential benefits of bottom-up approaches might have for addressing environmental problems.

**Fig. 5:**
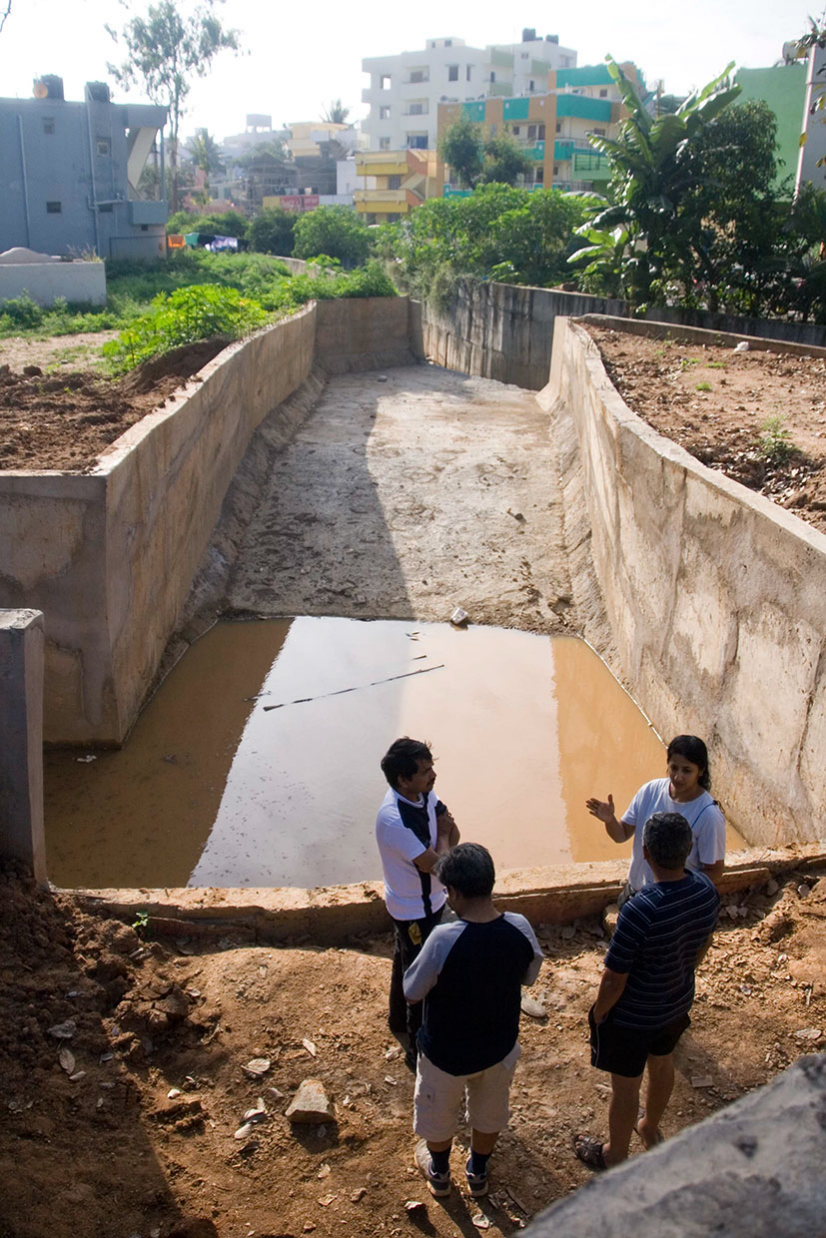
Members of a lake group inspecting the restoration work done by city authorities to regulate the flow of water from one of the group’s lakes to the one downstream (photo by Johan P. Enqvist)

As shown in previous research, MOU groups have—in collaboration with municipal and sometimes non-governmental partners—achieved more successful protection and management of individual lakes, compared to previous efforts led by municipal, state or private actors alone (Nagendra and Ostrom 2014; Enqvist et al. 2016; Nagendra 2016). While these groups have transformed management and become widely influential among other citizens concerned with the city’s lakes (Luna 2014; Nagendra 2016; Murphy et al. 2019), they are not the direct source of the good fit observed in our analysis. Instead, this stems from lesser-known and more recently formed lake groups, who approach lakes as interlinked chains that need to be cared for in a coordinated manner (Fig. 5). They do this either on their own, or in collaboration with an MOU group. Both quantitative and qualitative findings indicate that MOU groups still play a crucial role in supporting this new direction. In addition to confirming previous proposals that bottom-up initiatives can improve spatial fit (Guerrero et al. 2015), our research also provides new insights about how this can happen: innovative groups with a new formalized mandate pilot novel approaches to ecosystem-based management for individual lakes, and then advocate for and support others’ efforts to work in a similar way also at the network level.

These findings rely on social–ecological network analysis (Bodin and Tengö 2012; Bodin et al. 2014) to show that lake groups and their lake engagement does not occur at random, but in a pattern that suggest efforts to consider lake connectivity and water regulation properties. This is not just an effect of the groups preferring to work with nearby lakes that just happen to be linked hydrologically, since this should have produced more groups working with unconnected lakes (building block *d*). Further, groups working with connected lakes (building block *c*) explicitly state in interviews that this is a conscious strategy and inter-lake connectivity is part of their concerns. This demonstrates the value of mixing quantitative social–ecological network modeling framework with qualitative analyses, helping us to interpret the study results in ways that either method would have had difficulties to achieve alone. That said, of Bengaluru’s almost 200 lakes, our analysis only includes the approximately 15% that have active and easily identifiable lake groups. We focused on revealing tendencies in how current actors align their engagement in lakes and collaborations with other groups, and how this might impact the fit with the lakes they work with. We believe that these tendencies can be reasonably assumed to prevail if more lakes were being actively managed; however, this warrants further research. As shown here, such change processes are also shaped by ecosystem’s structures and dynamics, which support calls for more comprehensive views of social–ecological opportunities for transformative agency and innovation (Lubell et al. 2017).

Our study adds to a growing body of literature on Bengaluru’s lake groups, confirming previous claims that new initiatives tend to approach lakes as something more complex and diverse than simply ‘water bodies’ or ‘city parks’ (Enqvist et al. 2016; Derkzen et al. 2017; Murphy et al. 2019). MOU groups are presenting an integrated understanding of lakes’ ecological functions, historical role as water harvesting infrastructure, recreational uses for a growing middle class, livelihood importance for less affluent residents, and cultural significance in traditional rituals. This contributes to weaving a richer protective narrative (sensu Ernstson and Sörlin 2009), which helps build broader societal legitimacy than top-down standardized approaches that have resulted in lake-by-lake management based on city authorities’ jurisdictions. Further, the lake groups’ activities provide an opportunity for broader awareness, appreciation, and stewardship of nature in the city and beyond (Andersson et al. 2017; Murphy et al. 2019).

Most cities do not have interconnected lakes like Bengaluru, but urban areas commonly have dispersed and fragmented green and blue spaces that are in various ways connected or disconnected by various ecological processes (Lundberg et al. 2008; Holt et al. 2012; Bergsten et al. 2014). Ongoing and future urbanization projected for south Asia and elsewhere in the global South is spreading a need for better tools to manage these ecosystems. In cities where urban growth often outpace municipalities’ planning capacity, the shelter, sanitation, livelihoods, and other services that urban green and blue infrastructures can provide play a particularly important role for human wellbeing (McDonald et al. 2011, 2014; McPhearson et al. 2016; Nagendra et al. 2018). The potential of letting ecosystem management be partly driven by bottom-up initiatives is therefore of great interest, but not unproblematic. Public programs to promote environmental ‘stewardship’ in the global North are often seen as a way to identify ecosystem managers among those who benefit from them (Svendsen and Campbell 2008; Buijs et al. 2017), but have also been criticized for marginalizing the interests of those not able or allowed to access community groups (Baviskar 2003), and for promoting a neoliberal agenda of reducing public responsibilities (Rosol 2012). However, we also note that the informal sector already plays a significant role in many urban areas in the global South (Kudva 2009; Kooy 2014; Millington 2018; Nagendra et al. 2018) and ought to be better understood. This study contributes to this agenda by empirical testing of a theoretically informed method that can facilitate critical examination of collaborative initiatives. Bengaluru’s lake groups provide a rich spectrum of informality, from citizen groups operating on their own to hybrid arrangements between semiprofessional groups and municipal counterparts. This may be particularly important in cities’ vast peri-urban areas, where—like in our case—municipal services and infrastructure may be underdeveloped, and rapid urban sprawl more scattered, leaving more space to set aside land and lakes for protection (Nagendra and Ostrom 2014). Lake groups operating in peripheral parts of Bengaluru have been able to take on larger lakes (groups S and U, Fig. 1) or entire lake chains (groups H and J, Fig. 5). Our findings therefore provide several useful entry points to continued investigation of involving informal actors in ecosystem and water resource management. This includes more in-depth understanding of the interplay between more and less formal actors and processes, and how both horizontal and vertical collaboration can matter for unlocking and spreading innovation.

Policies that encourage bottom-up initiatives can allow actors to dictate their own priorities, enhancing fit with social needs and improving the sustainability of these efforts (Brown 2003; Guerrero et al. 2015). In Bengaluru, a decentralized but interconnected system for lake governance (cf. polycentric governance, see Ostrom 2010; Nagendra and Ostrom 2014) has existed in various forms previously in history. While those institutions were rooted in unequal social structures (Srinivas 2001; Nagendra 2016), they might still hold potential for learning about managing and promoting lakes’ multiple functions in the current urban landscape.

## Conclusions

How can social–ecological fit be improved in cities, where land is modified beyond recognition and large numbers of people inhabit ecosystems often without understanding their dynamics? In Bengaluru, part of the answer appears to be “gradually, through collaboration, and from the bottom-up.” The good fit displayed in how lake groups engage with the city’s network of lakes is driven by the most recent generation of such groups; however, it builds on wisdom from previous initiatives and many years of efforts to protect the city’s water bodies. By pioneering participatory lake management from 2010 onward, the MOU groups have claimed a space for lake activism in the city where communication and shared learning among the growing number of groups became crucial. This space has allowed good social–ecological fit to develop, through MOU groups collaborating with newer groups to manage interlinked lakes, and supporting other groups doing so on their own.

While this innovation process has been driven by grassroots, public agencies remain important gatekeepers and enablers of it. Key officials have been pivotal for critical changes to occur, such as ceding some of the previously centralized control of the lakes and recognizing citizen groups as legitimate partners. Local actors are not necessarily better managers than public authorities—in the Bengaluru case, only a fraction of the city’s lakes are cared for by local residents. However, allowing for and supporting bottom-up initiatives can broaden the portfolio of management options and remedy problems originating in social–ecological misfit, such as damage from flooding or lost livelihoods due to dried up lakes. Given the uncertain effects of ongoing environmental crises locally and globally, it is important to understand the potential and effectiveness of such options—not the least, given the unprecedented levels of urbanization reaching the global South and the vast numbers of people exposed to risks associated with environmental variability.

## Electronic supplementary material

Below is the link to the electronic supplementary material.

Supplementary material 1 (PDF 242 kb)

## Footnotes

1 By ‘governance,’ we refer to the broader system of institutions and decision-making processes including laws and regulations, bureaucratic and other power structures, norms and language. ‘Management’ refers to the implementation of the decisions from the governance system.
