# Supplementary material for: Are bottom-up approaches good for promoting social–ecological fit in urban landscapes?
Source: Ambio. 2019 Mar 16;49(1):49–61. doi: 10.1007/s13280-019-01163-4 (PMC6888795; doi:10.1007/s13280-019-01163-4)
Supplement: Supplementary file 1 — Supplementary material 1 (PDF 242 kb) [file 13280_2019_1163_MOESM1_ESM.pdf]

Ambio

Electronic Supplementary Material

This supplementary material has not been peer reviewed.

Title: **Are bottom-up approaches good for promoting social–ecological fit in urban landscapes?**

Authors: Johan P. Enqvist, Maria Tengö, Örjan Bodin

Appendix S1. Social–ecological network theory and methodology

A1.1. Defining a social–ecological network

The methodology that underpins the analysis of social–ecological systems as networks is intertwined with the theory underpinning the study; additional details on both are therefore provided here.

A social–ecological network represents a landscape of humans and other natural elements as a set of actors and ecological components (Bodin and Tengö 2012). A key assumption is that these social and ecological nodes are linked in various ways. In the Bengaluru lake groups case, a social–ecological link refers to a group's relationship to a lake; we define the existence of a link as there being a lake group claiming to be actively engaged in protecting or managing a specific lake. After defining this central social–ecological interdependency, we need to define social and ecological nodes that occur and have an impact at the same scale. In this case, these are the lake groups and the lakes they work with, respectively. It should be noted that since these definitions are centered around social–ecological relations, the study excludes groups that are not working with a specific lake, and also the majority of Bengaluru's almost 200 lakes where no group could be identified.

Further, the social-to-social and ecological-to-ecological links should be defined in a way that make them relevant to the chosen social–ecological links (Bodin and Tengö 2012). Here, two social nodes are considered as linked if at least one lake group reports some form of two-way communication with the other. As specified in the main text, 'communication' is defined as having regular meetings, visits to each other's lakes, recent exchange of information or advice, and/or at least one individual being members of both groups. This information was gathered through open-ended questions where we allowed respondents to freely recall all interactions with other groups. We consider connections to exist even if only one party reports it; since some groups may have many "incoming" links (other groups reporting interaction with them), they might not recall/report all of them accurately – however, it is unlikely that a group will report interaction with another group without having

actually talked to them. The face-to-face data collection allowed us to ask follow-up questions about stated interactions and guide the respondents in accordance with how we defined inter-group communication for this study, to accurately determine whether any two groups should be considered as linked in the social–ecological network model.

Links between two ecological nodes are defined by water flow, since this is a central feature of the main function of the lake ecosystem, and something that lake groups have demonstrated an awareness of and concern for (Luna 2014; Enqvist, Tengö, and Boonstra 2016). In Bengaluru, actual water flow can vary between wet and dry seasons and years, and is also shaped by rapid urban development. The map used to identify lake connections (STUP Consultants Pvt. Ltd. 2011) is therefore treated as a best estimate of how the topography of the terrain directs where water flows, or would flow, provided that enough rain falls and no obstacles block the path. Our model only considers direct links; in a chain of three lakes, only the ones immediately up- and downstream from each other are considered connected.

A1.2. Defining and measuring social–ecological fit

In order to measure fitness in a social–ecological network, this study uses the concept of social–ecological building blocks, sometimes called "motifs", each representing certain configurations of nodes and links in a social–ecological network (Bodin and Tengö 2012; Bodin et al. 2016). Different building blocks can be theoretically associated to certain types of social–ecological fit, or misfit. For example, if two lake groups work in parallel with two lakes that are hydrologically connected, and the groups communicate with each other, that would create a "good fit" building block of four nodes: two social nodes connected to each other as well as to two ecological nodes, also interconnected (illustrated in Table 1 in the main text, building block *h*). Building blocks like this represent ways in which actors and components of an ecosystem are aligned (or not). Below, we provide a more detailed description of the four categories of building blocks mentioned in the main text.

Shared lake. If two lake groups work with the same lake, active communication between them makes a 'collaborative sharing' (building block *a*), while absent communication indicates a 'non-collaborative sharing' (building block *b*). The former indicates a better fit since communication facilitates coordination of groups' work to optimize outcomes for the lake ("shared management" in Guerrero *et al.*, 2015).

Multiple lakes. If a lake group works with two lakes, water flow between them indicates a 'lake chain' (building block *c*) while no flow means 'unconnected lakes' (building block *d*). A lake chain implies an interconnected *system* that would benefit from being managed as an integrated whole, therefore this building block means social–ecological fit.

Mediated access. The third category describes situations where a lake group is connected to lakes or other groups only indirectly via another, mediating group. In 'centralized communication' (building block *e*), a central group is connected to two other groups that are not directly interacting with each other. In 'supported lake group' (building block *f*) a lake group has an external contact that is not working with the same lake. In 'supported chain group' (building block *g*) a group working on a lake chain communicates with a second group working with neither of those lakes.

Parallel work. Building blocks *h-j* all involve two lake groups working with one lake each. In 'lake chain collaborations' (building block *h*) both the groups and lakes are interconnected. In 'lake chain without collaboration' (building block *i*) only the lakes are linked, and in 'communication over unconnected lakes' (building block *j*) only the groups are. Collaboration over connected ecological units (indicating good fit, building block *h*) or lack of such collaboration (indicating misfit, building block *i*) have been studied before (Bodin et al. 2016; Guerrero et al. 2015). Here, we are also interested in which groups interact even if they work on lakes that are not connected (building block *j*), since this informs us about broader communication patterns. Table 1 (see main text) also describes what our hypotheses mean for expected observations in the studied network. Building blocks that indicate good fit are expected to be overrepresented, and those associated with poor fit

are expected to be underrepresented (Hypothesis 1). Similarly, MOU groups are expected to be present in 'good fit' building blocks and absent from 'poor fit' ones (Hypothesis 2).

Hypothesis 3 concerns the relationship between different types of lake groups and requires some additional explanation. To measure the impact of MOU groups on lake activism more broadly, this study categorizes all groups without MOUs into a 1st generation (groups founded before the MOUs were first introduced, in 2010) and a 2nd generation (groups founded after that event). Based on previous studies documenting the influential role of MOU groups on subsequent initiatives (Nagendra 2016; Enqvist, Tengö, and Boonstra 2016), we expect to see MOU groups centrally located in the network, connected to primarily 2nd generation groups (building block *e*). This also suggests that MOU groups to be the external contact supporting especially 2nd generation lake groups (building block *f*), including situations when these work on lakes that are not connected to the MOU group's lake (building block *j*). Together, this would confirm previous claims about MOU groups' influence on newer groups, but not inform whether this influence is associated to better fit. To do this, we study three other building blocks. First, we expect MOU groups to collaborate directly with newer, 2nd generation groups towards better fit, in 'collaborative sharing of lakes' (building block *a*), and in lake chain collaborations (building block *h*). Further, we expect MOU groups to mediate fit indirectly by being the external contact of 2nd generation 'supported chain groups' (building block *g*). This would all be strong indicators that MOU groups influence other groups to create better fit (Hypothesis 3). This study thus rests on the conceptualization of a social–ecological network consisting of a range of different building blocks. The ones chosen for analysis are selected to "capture theoretical assumptions on how certain links between and among actors and resources would be more or less desirable in enabling effective responses to different governance challenges" (Bodin et al. 2016, page 2). Identifying and mapping the frequencies of particular building blocks thus provides an entry point to defining and quantifying fit in the network. Further, by creating a large number of randomized networks of equal size (number of nodes and links) as the studied network, a null model is generated that is used as baseline to compare against the actual frequencies of the observed

building blocks in the system analyzed. This enables an assessment of whether any given building block is under- or overrepresented as compared to what would be observed purely at random. Previous applications of this methodology have identified building blocks with relevance for common pool resource management and for the alignment of social and ecological processes (Bodin and Tengö 2012; Kininmonth, Bergsten, and Bodin 2015; Guerrero et al. 2015), and also pointed to the potential of studying what individual actors most often occur in what building blocks (Bodin et al. 2014). Using this methodology we combine actor-level analysis and Guerrero et al.'s (2015) attention to bottom-up collaborative governance.

Having defined the network, and what constitutes fitness, we employed two tools to answer our hypotheses: a frequency analysis and a positional analysis. The frequency analysis informs us of the number of times each building block occurs in the observed network, compared to the distribution of said building block in a null (baseline) model (Bodin and Tengö 2012). The baseline was established using 1,000 randomized networks of the same size and structure (i.e. number of nodes and ties of each type) as the empirical network. The positional analysis (Bodin et al. 2014) informs us about which specific lake groups occur in what building blocks. This can be done both by comparing what groups are present in what building blocks, and where they are absent; another approach is to observe what positions they occupy in building blocks where there are two structurally different social nodes – e.g. building block *f* where a lake group is supported by communications with a group that does not work with that same lake (Table 1, main text). We use both of these approaches for this study.

References

Bodin, Örjan, and Maria Tengö. 2012. "Disentangling Intangible Social–Ecological Systems." Global Environmental Change 22 (2): 430–39. https://doi.org/10.1016/j.gloenvcha.2012.01.005.

Bodin, Örjan, Beatrice Crona, Matilda Thyresson, Anna Lea Golz, and Maria Tengö. 2014. "Conservation Success as a Function of Good Alignment of Social and Ecological Structures and Processes." Conservation Biology 28 (5): 1371–79. https://doi.org/10.1111/cobi.12306.

Bodin, Örjan, Garry Robins, Ryan R J McAllister, Angela M. Guerrero, Beatrice Crona, Maria Tengö, and Mark Lubell. 2016. "Theorizing Benefits and Constraints in Collaborative Environmental Governance: A Transdisciplinary Social-Ecological Network Approach for Empirical Investigations." Ecology and Society 21 (1). https://doi.org/10.5751/ES-08368-210140.

Enqvist, Johan, Maria Tengö, and Wiebren J. Boonstra. 2016. "Against the Current: Rewiring Rigidity Trap Dynamics in Urban Water Governance through Civic Engagement." Sustainability Science 11 (6): 919–33. https://doi.org/10.1007/s11625-016-0377-1.

Guerrero, Angela M, Örjan Bodin, Ryan R J McAllister, and Kerrie A Wilson. 2015. "Achieving Social-Ecological Fit through Bottom-up Collaborative Governance: An Empirical Investigation." Ecology and Society 20 (4).

Kininmonth, Stuart, Arvid Bergsten, and Örjan Bodin. 2015. "Closing the Collaborative Gap: Aligning Social and Ecological Connectivity for Better Management of Interconnected Wetlands." Ambio 44 (1): 138–48. https://doi.org/10.1007/s13280-014-0605-9.

Luna, Flor. 2014. "Transformation of Urban Lake Governance in Bangalore, India." Stockholm University, Sweden.

Nagendra, Harini. 2016. Nature in the City: Bengaluru in the Past, Present, and Future. New Delhi, India: Oxford University Press.

STUP Consultants Pvt. Ltd. 2011. "Map Showing Lakes in Bengaluru City (Entire BDA Area)." Bangalore, Karnataka, India: STUP Consultants Pvt. Ldt.

Appendix S2. Additional findings from network analysis

Several details in the study's findings are left out of the main text as they do not directly inform the hypotheses; however, we chose to include them here as they can still help us understand the dynamics relating to bottom-up emergence of social–ecological fit. Figure S2.1 shows the output data from the frequency analysis (summarized in Fig. 3 in the main text); Table S2.1 shows the data from the positional analysis (Fig. 4 in the main text).

A first observation relates to the absence of 'shared lakes' building blocks. Neither 'collaborative'/'non-collaborative sharing of a lake' (building blocks *a* and *b*) can be observed in the network, indicating that lake groups only work with lakes that no other group has already 'claimed'. This draws attention to a possible limitation in our methods: if one active group was identified for a lake, we did not continue searching independently for other ones. It is theoretically possible that more than one group was active on the same lake; however, no such situation was mentioned in any of the interviews either with lake groups or other key informants.

As for 'lake chain' building blocks, this category is dominated by group J, which works with a chain of five interconnected lakes (Fig. 2a in the main text). It occurs four times as a 'lake chain' group (building block *c*), but six times as a 'group with unconnected lakes' (building block *d*) (Table S2.1). This is a result of our strict definition of ecological links: in a chain of five lakes, lake 1 is not directly linked to lake 3, 4, and 5, lake 2 is not connected to lake 4 and 5, and lake 3 is not connected to lake 1 and 5, etc. In reality, group J's are all part of the same chain; if this group's presence in building block *d* is disregarded, this block is almost non-existent in the network, further strengthening the evidence of a good fit.

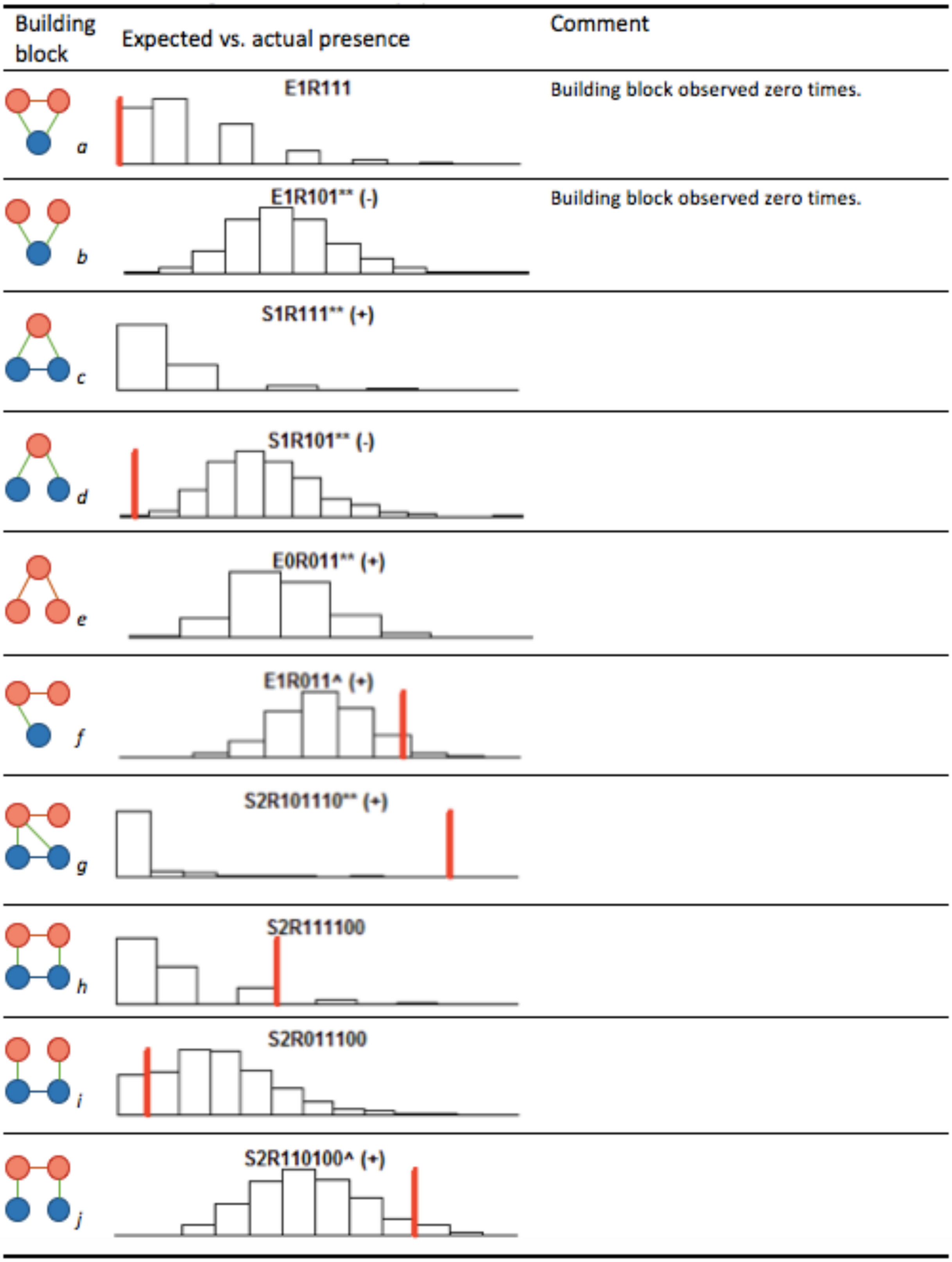

Figure S2.1 Observed number of building blocks (red lines) in relation to the normal distribution of said building block in randomized networks (bars). +/– indicate over- and underrepresentation (^ = weak significance ($p<0.01$), ** = strong significance ($p<0.05$)). This is the data behind Fig. 3 in the main text.

Table S2.1 Each line shows the presence of individual lake groups (A-W) in the studied building blocks and positions (columns a-j), as observed in the studied network. This is the data behind Fig. 4 in the main text.

	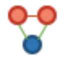	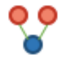	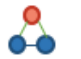	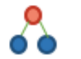	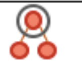	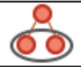	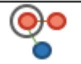	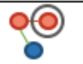	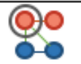	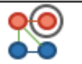	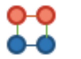	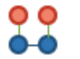	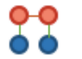
	a	*b*	*c*	*d*	*e-1*	*e-2*	*f-1*	*f-2*	*g-1*	*g-2*	*h*	*i*	*j*
A	0	0	0	0	0	0	0	0	0	0	0	0	0
B	0	0	0	0	0	0	0	0	0	0	0	1	0
C	0	0	0	0	0	0	0	0	0	0	0	0	0
D	0	0	0	0	0	0	0	0	0	0	0	0	0
E	0	0	0	0	0	0	0	0	0	0	0	0	0
F	0	0	0	0	0	9	1	2	0	0	0	0	2
G	0	0	0	1	45	8	20	15	0	5	1	0	29
H	0	0	1	0	1	16	4	3	2	0	0	0	6
I	0	0	0	0	26	6	8	9	0	1	0	0	9
J	0	0	4	6	1	11	10	3	8	0	0	0	15
K	0	0	0	0	0	7	1	1	0	0	0	0	1
L	0	0	0	0	0	0	1	1	0	0	1	0	0
M	0	0	0	0	0	8	2	2	0	0	0	0	2
N	0	0	0	0	4	15	4	5	0	0	0	0	5
O	0	0	0	0	1	16	2	3	0	0	0	0	3
P	0	0	0	0	0	0	1	1	0	0	1	0	0
Q	0	0	0	0	2	9	3	7	0	4	0	0	7
R	0	0	0	0	0	9	1	2	0	0	0	0	2
S	0	0	0	0	1	16	2	3	0	0	0	0	3
T	0	0	0	0	0	9	1	2	0	0	1	0	1
U	0	0	0	0	0	9	1	2	0	0	0	1	2
V	0	0	0	0	0	0	0	0	0	0	0	0	0
W	0	0	0	0	1	16	2	3	0	0	0	2	3

Our findings do not explore in depth why the 'lake chain groups' (H and J) choose to work with more than one lake. Both were formed around the time of the first MOU groups, and the fact that they are older than all other 2nd generation groups (Fig. 2 in the main text) could be viewed as an explanation for why they work with more lakes. However, interviews show that the groups have focused on the same lakes since the start. Another explanation could be that these lakes are located in a peripheral area where urban development is less intensive. With fewer immediate threats to the lakes, it might be easier to work with a whole chain. Both groups H and J are also well-connected and have (in addition to MOU groups) also interacted with different experts, local politicians, and NGOs.

Other findings also indicate that peripheral location can create different circumstances. Compared to the other MOU groups, Group L is not well known, it interacts with very few groups and does not support fit by interacting with a lake chain group. This could be related to its geographical location further away from central Bengaluru and having been active for a shorter period of time, compared to groups G and I (Fig. 2 in the main text). However, group L still contributes to social–ecological fit by being part of a 'lake chain collaboration' (building block *h*) with group P. The interview with the leader of group L also revealed a clear vision of decentralized lake management that acknowledges ecological links:

> Citizens' participation is a must. […] Government doesn't have funds; even if they have, it is not being used properly. […] According to me they should make about five or ten [lake modules, where each one] consists of five lakes. One person should be in charge and one trust [of local residents] should be in charge. Government should monitor it. [And you] make about five or ten modules, [and] connect them. [This way we could have the lakes of the city] completely restored, rejuvenated, developed and maintained within a span of three years.
>
> *Group L*

In addition to the network-related differences between the 1st and 2nd generation groups, interviews also indicate they vary in their objectives with their work. Newer groups more often talk about an "ecosystem approach" or "original design" to guide the lake restoration. This refers to the approaches advocated by the MOU groups, where lakes are restored with an explicit acknowledgment of the diverse functions and uses they used to have in a pre-urban landscape with seasonally fluctuating precipitation. Many of the 2nd generation groups have visited or plan to visit the lakes that MOU groups work on. Older groups tend to be less specific, primarily expressing a general desire to protect or rejuvenate the lake as a place of natural beauty.

Group H is the only 1st generation group working with a chain of lakes, which could be an example of the enabling role of lake custodians. The group has a very good collaboration with the local officer at the custodian authority BDA, including a high degree of influence over the management of the two lakes it works with. BDA has not (yet) adopted the approach of formally recogniz-

ing these collaborations in MOUs, but group H did not seem to need it. Other interviewees often described BDA as less forthcoming compared to BBMP; experiences differ depending on what civil servant is in charge of a group's area.

At BBMP, at least two individuals that are particularly forthcoming; one more senior, working to advocate for lakes within the bureaucracy, the other more in direct contact with many of the lake groups. The latter is mentioned by virtually all groups with BBMP lakes, including those with MOUs, as remarkably supportive. Lake groups as well as this official himself pointed out that allowing for decentralization of power is quite uncommon in Bengalurean (if not Indian) public administration.
